# Repeated injections of _D_-Amphetamine evoke rapid and dynamic changes in phase synchrony between the prefrontal cortex and hippocampus

**DOI:** 10.3389/fnbeh.2013.00092

**Published:** 2013-07-30

**Authors:** Sungwoo Ahn, David N. Linsenbardt, Christopher C. Lapish, Leonid L. Rubchinsky

**Affiliations:** ^1^Department of Mathematical Sciences, Center for Mathematical Biosciences, Indiana University Purdue University IndianapolisIndianapolis, IN, USA; ^2^Department of Psychology, Indiana University Purdue University IndianapolisIndianapolis, IN, USA; ^3^Stark Neurosciences Research Institute, Indiana University School of MedicineIndianapolis, IN, USA

**Keywords:** amphetamine, hippocampus, local field potential, oscillations, phase-locking, prefrontal cortex, synchrony

## Abstract

Repeated drug use evokes a number of persistent alterations in oscillatory power and synchrony. How synchronous activity in cortico-hippocampal circuits is progressively modified with repeated drug exposure, however, remains to be characterized. Drugs of abuse induce both short-term and long-term adaptations in cortical and hippocampal circuits and these changes are likely important for the expression of the altered behavioral and neurobiological phenotype associated with addiction. The present study explores how the initial (up to 1 h) pharmacological response to _D_-Amphetamine (AMPH) is altered with repeated injections in the rat. The methods employed herein allow for the progressive changes in synchronized dynamics with repeated intermittent AMPH exposure to be characterized over short time scales (minutes). Specifically, we examined the temporal variations of phase-locking strength in delta and theta bands within the prefrontal cortex (PFC) and between PFC and hippocampus (HC) shortly after drug injection. After the first injection of AMPH synchrony increased within the PFC in the delta band, which was followed, by an increase in theta synchrony between the PFC and HC several minutes later. This relationship switched after repeated AMPH injections, where increases in theta synchrony between the PFC and HC preceded increases in delta synchrony in the PFC. The time-course of increases in synchronous activity were negatively correlated between the PFC delta and the PFC-HC theta. Collectively these data highlight the potential role of PFC-HC circuits in the development of addiction and outline dynamic changes in the time-course that cortico-hippocampal circuits become synchronized with repeated AMPH exposure.

## Introduction

The progressive changes in behavior and underlying adaptations in neurobiology that occur after repeated psychostimulant exposure have been studied extensively, and are thought to underlie the increases in the incentive motivational properties of addictive drugs following repeated use (Robinson and Berridge, 1993). However, understanding how repeated drug administration leads to altered function in localized and distributed neural systems is still forthcoming, and may be of particular importance since the expression of drug seeking behaviors depends on the interaction of numerous brain regions (McFarland et al., 2003; Sun and Rebec, 2003).

Oscillations facilitate a number of phenomena throughout the brain (Buzsáki and Draguhn, 2004) including neural communication (Varela et al., 2001), the formation of cell assemblies (Benchenane et al., 2010), memory formation (Fell and Axmacher, 2011), and plasticity (Chauvette et al., 2012). Furthermore, abnormal oscillatory activity is also observed in a number of disease states, including addiction (Schnitzler and Gross, 2005; Reid et al., 2006; Uhlhaas and Singer, 2006, 2010). Alterations in EEG activity are commonly observed under the acute influence of abused drugs and also following recent abstinence from abused drugs (Newton et al., 2003; Saletu-Zyhlarz et al., 2004; Greenwald and Roehrs, 2005; Herning et al., 2005; Reid et al., 2006). Changes in oscillatory activity are also observed for some time after cessation of drug use across multiple frequency bands and brain regions (Herning et al., 1994; Newton et al., 2003; Reid et al., 2003, 2008). Animal models of addiction also exhibit persistent changes in oscillatory power and synchrony (Lapish et al., 2012a; Ahn et al., 2013), suggesting that further exploration of these phenomena has the potential to inform our understanding of how systems level neural communication is altered by repeated drug use. Understanding oscillatory dynamics also has translational potential as they are experimentally accessible metrics that can be assessed in both addicted individuals and rodent models of this condition.

Converging evidence suggests that neuroadaptations in biology (Gipson et al., 2013) and physiology (Lapish et al., 2012a) occurring in the prefrontal cortex (PFC) with repeated drug use are particularly robust and are associated with the probability of relapse (Bauer et al., 1994, 2001; Winterer et al., 1998). As such, understanding how repeated drug use alters neural communication within the PFC and the brain regions it interacts with may provide critical insights into how information processing is altered in addiction. Synchronous activity between the PFC and hippocampus (HC) has been shown to be particularly robust and is necessary for a variety of cognitive processes (Hyman et al., 2005; Jones and Wilson, 2005; Siapas et al., 2005). Furthermore, synchrony between these regions is altered after drug exposure in humans (Reid et al., 2008) and rodents (Dzirasa et al., 2006; Lapish et al., 2012a; Ahn et al., 2013) and therefore may be critical for the expression of addictive behaviors.

Synchronous activity between oscillators is influenced by fluctuations in both the amplitude and phase domain. However, as oscillators become coupled, synchrony in the phase domain generally emerges prior to amplitude correlations (Pikovsky et al., 2001). Thus, assessing phase synchrony provides a sensitive metric to detect the progressive changes in synchrony that develops as the strength of coupling between oscillators increases (Lachaux et al., 1999; Pikovsky et al., 2001; Hurtado et al., 2004; Le Van Quyen and Bragin, 2007; Ahn et al., 2013). In particular, alterations in phase synchrony evoked by AMPH are observed prior to those in the amplitude domain (Lapish et al., 2012a; Ahn et al., 2013) thus recommending further exploration of phase synchronization phenomena to understand how neural circuits are progressively altered shortly after each repeated drug administration.

In the current study repeated injections of a single dose of AMPH were employed to assess the temporal characteristics of phase-locking of delta and theta band oscillations within the PFC and between the PFC and HC. The methods employed herein allow for the progressive within- and between- session changes in synchronized activity following AMPH treatment to be quantified and characterized. We reveal changes in PFC and HC circuits to AMPH administration as these responses develop over the time-course of few minutes. The results of the study emphasize the dynamic evolution of AMPH-induced synchronous activity over short time scales (minutes) in response to each drug administration.

## Materials and methods

### Animals

A total of 8 male Long-Evans rats were purchased from Charles River (Saint-Constant, QC), given one week of acclimation to single housing and vivarium conditions, and then implanted with microelectrode arrays. Animals had *ad lib* access to food and water except during behavioral testing/electrophysiological recording sessions. All animals were treated in accordance with the ethical standards outlined by the CCAC and IACUC.

### Electrodes

For an in depth descriptions of electrophysiological and behavioral procedures see Lapish et al. (2012a). Briefly, electrodes were fabricated in house by feeding 25 μM tungsten wires (California Fine Wires, Grover Beach, CA) through matrices of polyamide coated compressed silica tubing yielding recording electrodes separated by ~150 μM. Electrode wire matrices were then secured to an electronic interface board (EIB-27; Neuralynx, Bozeman, MT) with epoxy. A total of 16 electrodes arranged in a 2 × 8 array were implanted in the PFC and a total 4 electrodes arranged in a 1 × 4 array were implanted in the HC.

### Surgery

Electrode arrays were unilaterally implanted lengthwise in the anterior/posterior axis of the medial PFC (centered on AP +3.2, ML +0.5, DV −3.0, relative to bregma) and dorsal HC (AP −3.6, ML +2.0, DV −2.5, relative to bregma) as described in Lapish et al. (2012a). While animals were under isoflurane anesthesia probes were lowered into the targeted brain region and fixed in place with dental acrylic. Each animal was given a minimum of one week for post-operative recovery before experiments were performed.

### Repeated AMPH administration procedures

All animals were first attached to the recording tether and placed into a 55 cm in diameter white Plexiglas open field arena surrounded by a 20 cm white Plexiglas wall and allowed to freely explore for ~15 min while electrophysiological activity was recorded. Animals then received a single intra-peritoneal injection of either 1.0 mg/kg AMPH (*N* = 4) dissolved in 0.9% saline or an isovolumetric dose of saline (*N* = 4) and were immediately placed back into the open field arena where recording continued for ~45 additional minutes. These procedures were repeated every other day for the first 9 days of the experiment yielding a total of five injections. Following this “induction phase” animals were given a 14 day “incubation period” during which time the animals remained in their home cages and were otherwise not handled or manipulated except for weekly cage changes. After this time, animals were given a single injection of AMPH in an identical manner as described in the induction phase: animals were placed in open field, given an AMPH or saline injection, and electrophysiological activity was recorded.

### Electrophysiology recording parameters

All electrode wires were externally referenced to a stainless steel skull screw over the cerebellum. Local field potentials (LFPs) were acquired with a 24 channel Neuralynx Cheetah recording system, amplified 2000 times, and sampled at 30303 Hz. Recordings were filtered between 0.01 and 1000 Hz and then resulting signals were down sampled to 947 Hz off line for analysis. The proper placement of recording electrodes was confirmed via histology.

### Phase-locking analysis

**Figure 1:**
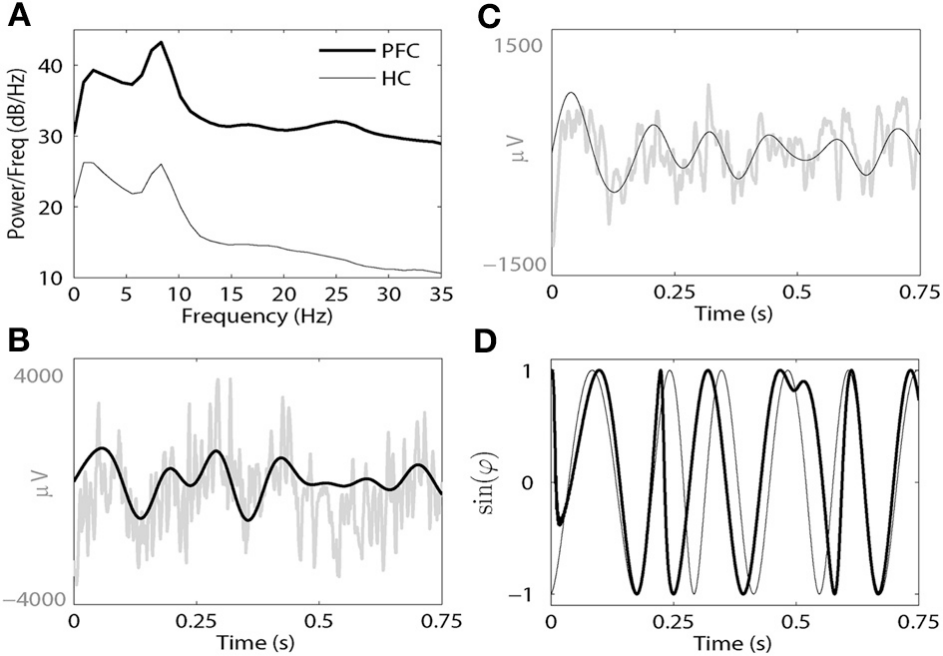
**(A)** A representative power spectral density (PSD) plot from the PFC (thick black line) and HC (thin black line) from a single animal following AMPH injection on Day 9. **(B)** Raw (gray line) and filtered LFPs from the PFC (thick black line) at the theta band. **(C)** Raw (gray line) and filtered LFPs from the HC (thin black line) at the theta band. **(D)** The sine functions of the phases of both filtered signals (thick line for the PFC and thin line for the HC).

Signals from all electrodes were Kaiser windowed and digitally filtered with an FIR filter in delta (2.5 ~ 5 Hz) and theta (5 ~ 11 Hz) bands. Zero-phase filtering was used to avoid phase distortions. Phase was extracted via Hilbert transform resulting in two signals; ϕ_1_(*t*) and ϕ_2_(*t*) (see Pikovsky et al., 2001; Hurtado et al., 2004). Examples of filtered signals and resulting phases are presented in Figure 1. The following standard measure of the strength of phase locking between these two signals was calculated:

$$\gamma ={\left\|\frac{1}{N}\sum_{j\;=\;1}^{N}{\text{e}}^{i\theta (t_{j})}\right\|}^{2},$$

where θ(*t*_*j*_) = ϕ_1_(*t*_*j*_) − ϕ_2_(*t*_*j*_) is the phase difference, *t*_*j*_ are the times of data points, and *N* is the number of all data points during the given time interval. This phase-locking index results in values that range from 0 (no phase locking) to 1 (perfect phase locking). This kind of phase synchrony index has been shown to be sensitive and appropriate to study neural oscillatory synchronization of widely varying strength (Lachaux et al., 1999; Pikovsky et al., 2001; Hurtado et al., 2004). The phase extraction and methods for computing γ are illustrated in Figure 1. Figure 1A illustrates the power spectral density (PSD) of unfiltered signals from the PFC (thick line) and HC (thin line) following an AMPH injection in a single animal. Note that in each brain region there is a prominent peak in both delta and theta bands. Figures 1B,C present examples of unfiltered and filtered LFP from PFC (Figure 1B) and HC (Figure 1C) in the theta band. Figure 1D is an illustration of the sine functions of the extracted phases from these two brain regions.

**Figure 2:**
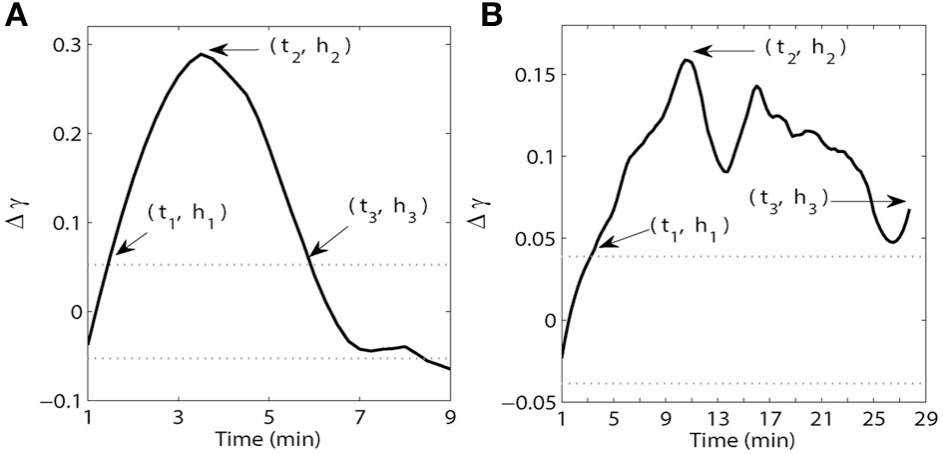
**Examples of time and magnitude measures (*t*_*i*_, *h*_*i*_) for synchrony within the PFC (A) and between the PFC and HC (B).** The solid line is the synchrony strength Δγ(*t*) after smoothing with a third order Savitzky-Golay filter. The dotted lines indicate 95% confidence intervals of the phase-locking index γ during the pre-injection period for each pair of wires minus γ_pre_ where γ_pre_ is the mean value of the phase-locking index γ during the pre-injection period.

In order to assess how synchronous oscillatory activity changes over time, we computed the phase-locking index γ over non-overlapping 3 min recording windows. We then used the 3 min window immediately prior to the injection and then 9 non-overlapping 3 min windows following injection. To determine the relative change in synchrony following injection, we computed the difference of the phase-locking indices between pre- and post-injection windows for each electrode wire. That is,

$$△\gamma (t_{j})=\gamma_{\text{post}}(t_{j})-\gamma_{\text{pre}},$$

where *t*_*j*_ = {3, 6, 9, 12, 15, 18, 21, 24, 27} min, γ_pre_ is the phase-locking index of the last 3 min prior to the injection, γ_post_(*t*_*j*_) is the phase-locking index for each time interval during the post-injection period. The difference in synchrony levels Δγ(*t*_*j*_) are used to detect if the effect of injection is significant. If the effect is significant, we determined the onset, peak, and duration of the effect with high temporal resolution. Thus, we considered running windows of 1 min duration shifted by 0.25 min. To study the time-course of synchrony (relative to pre-injection epoch), we computed the difference between the phase-locking indices γ_post_(*t*_*j*_) at *t*_*j*_ = {1, 1.25, 1.5, 1.75, 2, …} min and the mean phase-locking index of pre-injection epoch γ_pre_. The resulting time-series of Δγ(*t*) was smoothed with a third order Savitzky-Golay filter (Orfanidis, 1996). Smoothing the curves with this filter allowed small and inconsistent changes to be removed in order to detect general features of the response (see next paragraph). Significant increases in synchrony during the post-injection epoch were evaluated by determining if and when synchronous activity crossed the 95% upper confidence interval (CI) of the phase-locking index γ during the pre-injection period (see Figure 2). If the smoothed Δγ(*t*) was higher than this threshold for at least 3 min, then the crossing point was considered as the start of the elevated synchrony.

**Figure 3:**
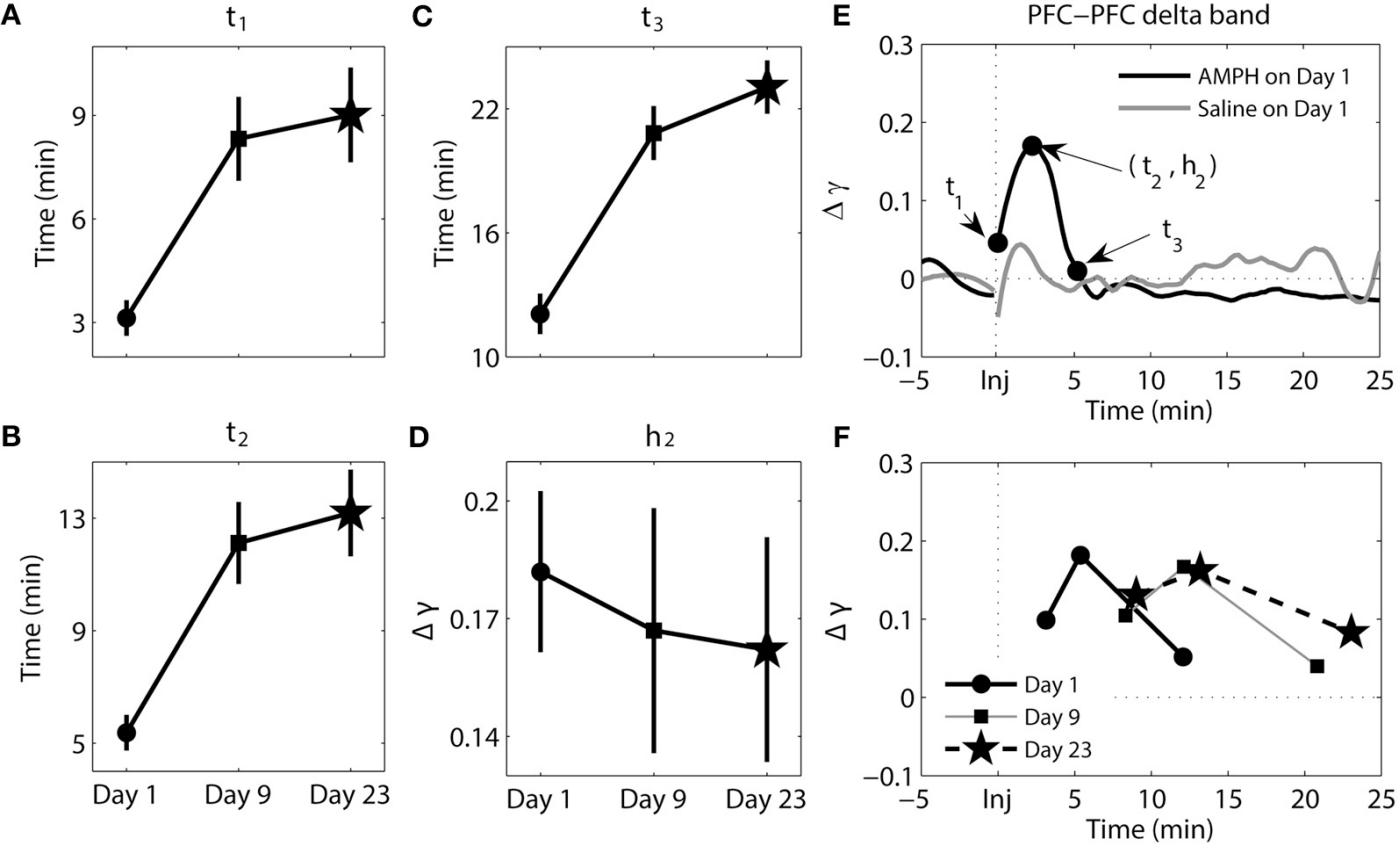
**Dynamics of synchrony within the PFC at the delta band in response to AMPH injection. (A–D)** Time and magnitude measures (*t*_1-3_, *h*_2_) for different days (Day 1 is the day of the first injection, Day 9 is the day of the last injection prior to the 2-week incubation period, and Day 23 is the day of the challenge test). **(E)** Examples of time-courses of synchrony strength Δγ(*t*) for Saline and AMPH animals on Day 1 with (*t*_1-3_, *h*_2_). **(F)** The mean values of (*t*_i_, *h*_*i*_) on each day. Error bars in **(A–D)** indicate standard error of the mean (±SEM).

**Figure 4:**
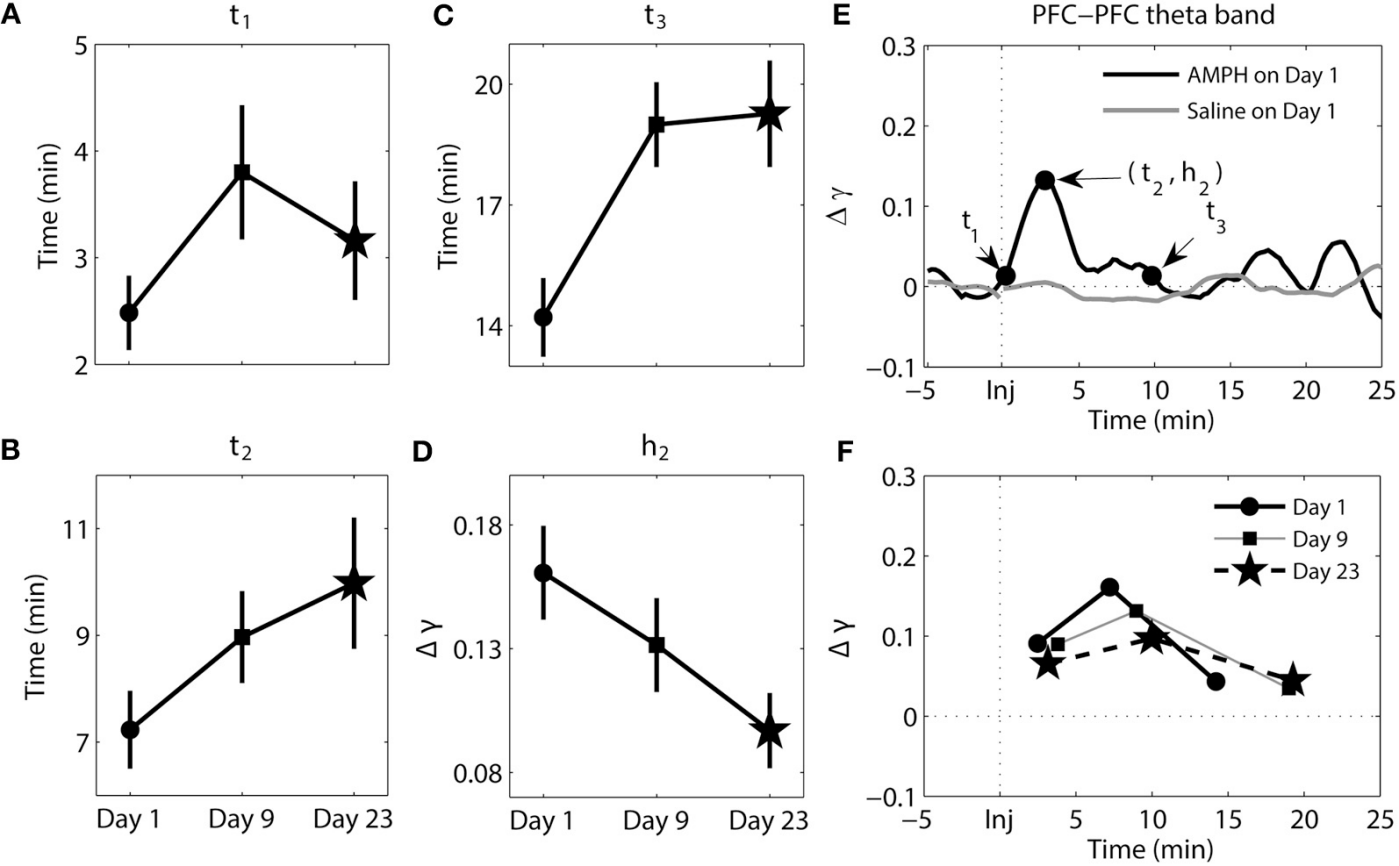
**Dynamics of synchrony within the PFC at the theta band in response to AMPH injection. (A–D)** Time and magnitude measures (*t*_1-3_, *h*_2_) for different days (Day 1 is the day of the first injection, Day 9 is the day of the last injection prior to the 2-week incubation period, and Day 23 is the day of the challenge test). **(E)** Examples of time-courses of synchrony strength Δγ(*t*) for Saline and AMPH animals on Day 1 with (*t*_1-3_, *h*_2_). **(F)** The mean values of (*t*_*i*_, *h*_*i*_) on each day. Error bars in **(A–D)** indicate standard error of the mean (±SEM).

**Figure 5:**
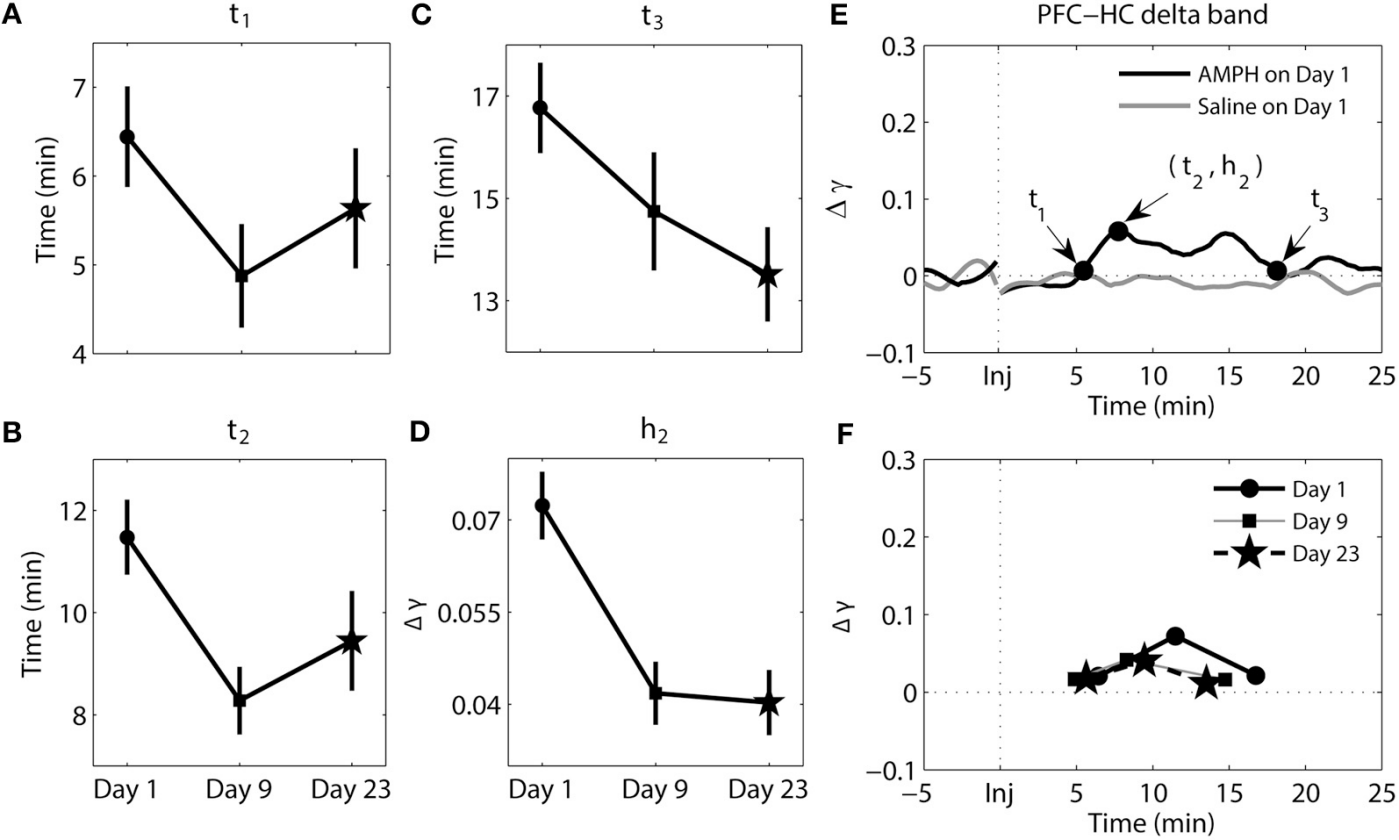
**Dynamics of synchrony between the PFC and HC at the delta band in response to AMPH injections. (A–D)** Time and magnitude measures (*t*_1-3_, *h*_2_) for different days (Day 1 is the day of the first injection, Day 9 is the day of the last injection prior to the 2-week incubation period, and Day 23 is the day of the challenge test). **(E)** Examples of time-courses of synchrony strength Δγ(*t*) for Saline and AMPH animals on Day 1 with (*t*_1-3_, *h*_2_). **(F)** The mean values of (*t*_*i*_, *h*_*i*_) on each day. Error bars in **(A–D)** indicate standard error of the mean (±SEM).

**Figure 6:**
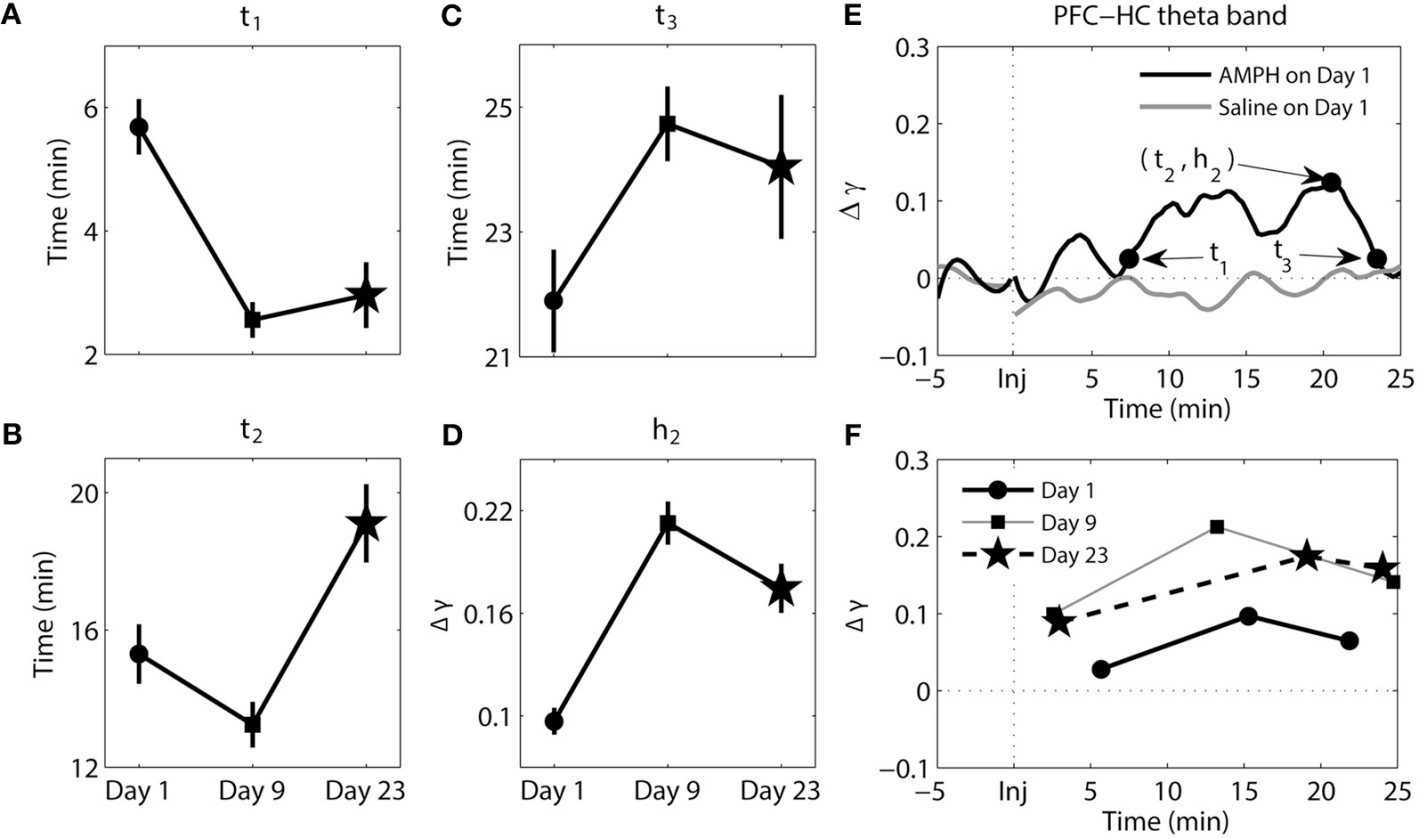
**Dynamics of synchrony between the PFC and HC at the theta band in response to AMPH injections. (A–D)** Time and magnitude measures (*t*_1-3_, *h*_2_) for different days (Day 1 is the day of the first injection, Day 9 is the day of the last injection prior to the 2-week incubation period, and Day 23 is the day of the challenge test). **(E)** Examples of time-courses of synchrony strength Δγ(*t*) for Saline and AMPH animals on Day 1 with (*t*_1-3_, *h*_2_). **(F)** The mean values of (*t*_*i*_, *h*_*i*_) on each day. Error bars in **(A–D)** indicate standard error of the mean (±SEM).

To characterize the time-course of synchrony we measured the timing and magnitude of three events. The onset of the increase in synchrony above the upper 95% CI of the baseline (pre-injection) level was determined and referred to as *t*_1_ (Figure 2). The time of peak (maximal) synchrony (*t*_2_) and the time synchrony stayed elevated before crossing back below the upper 95% CI of the pre-injection baseline (*t*_3_) were also determined. In instances where the effect remained above baseline for the duration of the recording session the final time point of the session was chosen as *t*_3_. The magnitude of synchrony (relative to pre-injection baseline level) at each time point *t*_1−3_ was also determined and was referred to as *h*_1−3_. In this way, we characterized the time-course of synchrony for each frequency band and brain region/circuit. Note that *h*_1_ and *h*_3_ are strongly influenced by pre-injection dynamics (as they are mostly defined by the variance of pre-injection synchrony), thus we opted to not use them for statistical analysis or for making any conclusions about the dynamics of post-injection synchrony. These purely qualitative measures are presented for illustrative purposes in Figures 3–6 subplots F. Each of these metrics are further described in Figure 2, which contains examples of the smoothed Δγ(*t*) for one AMPH animal obtained from two PFC recording sites as well as a recording from one PFC site and one HC site on Day 1 in the theta band. Following injection of AMPH, synchrony within the PFC quickly increased, peaked within a few minutes, and then quickly returned to pre-injection baseline levels (Figure 2A). In contrast, synchrony between the PFC and HC increased slowly, peaked several minutes after the peak observed within PFC, and then stayed above pre-injection baseline throughout the remainder of the session (Figure 2B).

Finally, we determined if significant differences in *t*_1−3_ or *h*_2_ existed between the two brain regions and how large these differences were. We first computed the average measures for each brain region/circuit separately, taking into account the region/circuit-specific number of possible electrode pair combinations. Within the PFC for each animal on each day, we computed an average time measure of elevated synchrony for *i*^th^ PFC electrode as:

$$t^{av(i)}=\frac{1}{7}\sum_{j\neq i}^{8}t(i,j),$$

where *t*(*i*, *j*) is the time measure (*t*_1−3_) of elevated synchrony between *i*^th^ and *j*^th^ electrodes in the PFC. Similarly, for the PFC-HC circuit

$$t^{av(i)}=\frac{1}{4}\sum_{j=1}^{4}t(i,j),$$

where *t*(*i*, *j*) is the time measure (*t*_1−3_) of elevated synchrony between *i*^th^ PFC electrode and *j*^th^ HC electrode. We then averaged across all animals on each day to obtain eight *t*^*av*^ values for within the PFC and PFC-HC on each day. Synchrony magnitude *h*^*av(i)*^ and *h*^*av*^ were computed for each region on each day in a similar fashion.

### Statistics

Analysis was performed in MATLAB (Mathworks, Nautick, MA) and R (www.r-project.org/). Unless specified otherwise, all *t* and *h* variables were first subjected to repeated measures analysis of variance testing (ANOVA) with treatment group, injection epoch, and day as the factors. Tukey's *post-hoc* tests were used when appropriate. Significance level was set at α = 0.05.

## Results

### Repeated AMPH evokes changes in the time-course of synchronized activity

To compare the effects of the injection (pre vs. post) and treatment group (Saline vs. AMPH) using the phase-locking index γ across 3 min time bins during the pre- and post-injection period, we first conducted a factorial ANOVA with treatment group and injection day as between groups factors and 3 min time bins as within subjects factor for each brain region separately. Within the PFC, a significant main effect of the injection day was observed [*F*_(9, 7636)_ = 9.493, *p* = 1.63e-14]. Between the PFC and HC, there were significant main effects of treatment group [*F*_(1, 8356)_ = 136.65, *p* ≤ 2.0e-16] and injection day [*F*_(9, 8356)_ = 22.331, *p* ≤ 2.0e-16], as well as a significant treatment group × injection day interaction [*F*_(9, 8356)_ = 2.921, *p* = 1.85e-3]. *Post-hoc* tests indicated that this interaction was driven by significant differences in synchrony between pre- and post-injection periods in the AMPH group (Tukey's *post-hoc* tests, *p* ≤ 4.13e-5) but not the Saline group (Tukey's *post-hoc* tests, *p* > 0.05). Thus, in both brain regions only AMPH treated animals had significant alterations in synchrony during the post-injection epoch. So, we focused this study on the analysis of AMPH animals only, with high temporal resolution for each brain region and frequency band on each day.

### The onset time, latency to peak, and duration of elevated synchrony increases in the PFC with repeated AMPH injections

The results of analysis of the delta band within the PFC can be seen in Figure 3. For this Figure and Figures 4–6, panels **A–D** illustrate each variable separately. However, to provide an integrated view of how each of variables changes with repeated injection, the means are plotted together in panel **F**. In Figures 3–6 panel **E**, examples of time course of synchrony strength Δγ(*t*) are plotted for both Saline and AMPH animals on Day 1. There were significant main effects of day for all three time measures [*t*_1_ − *F*_(2, 154)_ = 11.78, *p* = 1.74e-5; *t*_2_ − *F*_(2, 154)_ = 15.19, *p* = 9.53e-7; *t*_3_ − *F*_(2, 154)_ = 27.84, *p* = 4.78e-11]. *Post-hoc* testing confirmed that it was due to significantly lower values on Day 1 compared to both Day 9 (Tukey's *post-hoc* tests, *p* ≤ 1.35e-3) and Day 23 (Tukey's *post-hoc* tests, *p* ≤ 5.77e-5). This effect illustrates the delay in the peak of synchrony on Day 9 and Day 23 compared to Day 1; synchrony significantly increased, peaked, and went back to baseline more quickly on Day 1 following the first AMPH experience (Figure 3F). There was no significant difference in the magnitude of synchrony *h*_2_ (Tukey's *post-hoc* tests, *p* > 0.05).

The results of analysis of the theta band within the PFC can be seen in Figure 4. There were significant main effects of day for *t*_3_ [*F*_(2, 207)_ = 7.25, *p* = 9.03e-4] and *h*_2_ [*F*_(2, 207)_ = 3.10, *p* = 4.71e-2]. *Post-hoc* tests confirmed that *t*_3_ values were significantly smaller on Day 1 compared to Day 9 (Tukey's *post-hoc* tests, *p* = 6.33e-3) and Day 23 (Tukey's *post-hoc* tests, *p* = 3.45e-3); synchrony went back to baseline more quickly on Day 1. For *h*_2_, *post-hoc* tests confirmed that peak synchrony on Day 1 was significantly higher than peak synchrony on Day 23 (Tukey's *post-hoc* tests, *p* = 3.63e-2).

Collectively these data highlight that with repeated injections of AMPH the onset of elevated synchrony, latency to peak synchrony, and duration of elevated synchrony in PFC delta (and to a degree in theta) become progressively longer after injection.

### The onset of elevated synchrony decreases while magnitude of synchrony increases between the PFC and HC with repeated AMPH injections

The results of analysis of the delta band between the PFC and HC can be seen in Figure 5. There were significant main effects of day for *t*_2_ [*F*_(2, 193)_ = 3.97, *p* = 2.04e-2], *t*_3_ [*F*_(2, 207)_ = 3.21, *p* = 4.27e-2], and *h*_2_ [*F*_(2, 193)_ = 11.81, *p* = 1.46e-5]. Whereas the magnitude of synchrony (*h*_2_) was higher on Day 1 compared to Day 9 (Tukey's *post-hoc* tests, *p* = 6.16e-4) and Day 23 (Tukey's *post-hoc* tests, *p* = 9.06e-5), the time at which peak synchrony occurred (*t*_2_) was only different between Day 1 and Day 9 (Tukey's *post-hoc* tests, *p* = 2.17e-2). A significant difference at *t*_3_ was observed between Day 1 and Day 23 (Tukey's *post-hoc* tests, *p* = 3.73-2). However, overall the change in phase synchrony (Δγ) in this band between these two structures was very weak. In the analysis of synchrony within PFC in theta and delta bands and between PFC and HC in theta band Δγ were approximately two-three times of magnitude larger than Δγ for delta band PFC-HC synchrony (compare Figures 3F, 4F, 6F with 5F).

The results of analysis of the theta band synchrony between the PFC and HC can be seen in Figure 6. There were significant main effects for all measures of *t* [*t*_1_ − *F*_(2, 269)_ = 17.03, *p* = 1.09e-7; *t*_2_ − *F*_(2, 269)_ = 10.83, *p* = 2.99e-5; *t*_3_− *F*_(2, 269)_ = 3.24, *p* = 4.08e-2] and *h*_2_ [*F*_(2, 269)_ = 26.87, *p* = 2.30e-11]. It took significantly longer for elevated synchrony to set in after AMPH injection on Day 1 compared to AMPH injection on Day 9 (Tukey's *post-hoc* tests, *p* = 3.36e-7) and Day 23 (Tukey's *post-hoc* tests, *p* = 3.93e-5). On the contrary, peak synchrony (*t*_2_) on Day 23 was reached significantly later than Day 1 (Tukey's *post-hoc* tests, *p* = 8.97e-3) and Day 9 (Tukey's *post-hoc* tests, *p* = 1.69e-5). Difference at *t*_3_ was significant between Day 1 and Day 9 only (Tukey's *post-hoc* tests, *p* = 3.823-2). Significant difference in synchrony magnitude measure *h*_2_ was observed: *h*_2_ was significantly lower on Day 1 than on Day 9 and Day 23 (Tukey's *post-hoc* tests, *p* ≤ 2.64e-5).

In sum, while changes in the pattern of synchrony between the PFC and HC in response to each AMPH injection were observed in the delta band, these values were much smaller than those in theta band; meaning synchrony between the PFC and HC was weak in the delta band. In the theta band, increases in synchrony were much more substantial. They occurred quicker but took longer to peak and subsequently decayed with repeated injections of AMPH. Moreover, peak synchrony dramatically increased in response to AMPH injections on Day 9 and Day 23 compared with Day 1.

### A negative correlation in the onset of elevated synchrony within the PFC and between the PFC-HC is observed with repeated AMPH injections

**Figure 7:**
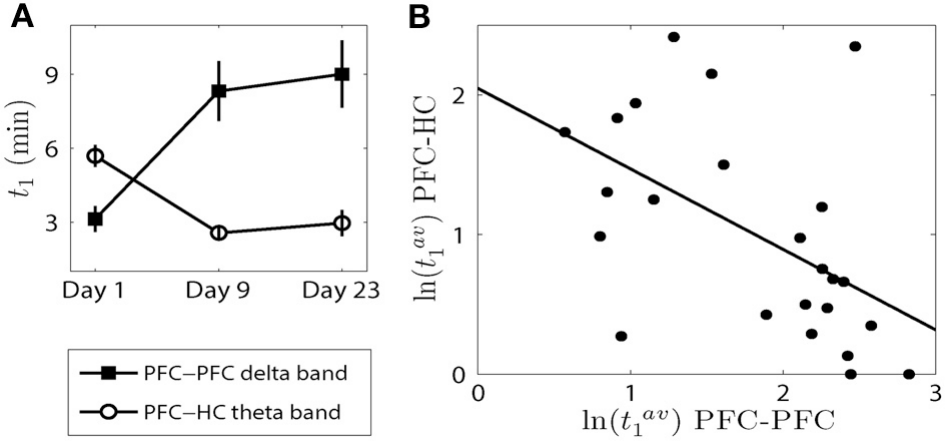
**The onset of elevated synchrony between the PFC-PFC delta band and PFC-HC theta band are negatively correlated. (A)** The means and SEMs of the onset of elevated synchrony *t*_1_ on each day (c.f. Figures 3A, 6A). **(B)** Scatter plot with the best linear fit shows the time difference of *t*_1_ (in logarithmic scale) on each day within the PFC delta band and in the PFC-HC theta band.

In this subsection we compare how the elevated synchrony changes from day to day between the PFC-PFC delta band and the PFC-HC theta band. We observed that there were significant main effects of day for all three time measures within the PFC delta band and between the PFC and HC theta band (see Figures 3, 6). We examined the time difference in the onset of synchrony *t*_1_ within the PFC delta band and between the PFC-HC theta band because the results of analysis presented above pointed to the most significant change in the elevated synchrony onset times for these two cases. A significant main effect of brain region was observed [*F*_(1, 42)_ = 21.55, *p* = 3.37e-5; Figure 7A] and a robust brain region × day interaction was observed [*F*_(2, 42)_ = 15.65, *p* = 8.33e-6; Figure 7A]. This was further explored in the correlation of average elevated synchrony onset times *t*^*av*^_1_ between the PFC-PFC delta band and the PFC-HC theta band (see Materials and Methods). There was a strong negative correlation between logarithmic transformations of *t*^*av*^_1_ values (*R* = −0.5267, *p* = 8.19e-3; Figure 7B) pointing to a significant negative correlation of elevated synchrony onset times between the PFC-PFC delta band and PFC-HC theta band. Thus, a synchronous response to the first injection of AMPH occurred first within the PFC delta band and was followed by the PFC-HC theta band several min later. In contrast to this, AMPH injections on Day 9 and Day 23 first led to a response in the PFC-HC theta band, followed by the onset of delta band elevated synchrony within the PFC.

### Changes in the time course of synchrony are not attributable to pre-injection variance

To assess the contribution of variance across days in the pre-injection epoch on *t*_1−3_ and *h*_2_, we took the variance of each pre-injection epoch into account when setting the threshold for an increase in synchrony. The synchrony values during pre-injection epochs on all three different days for all animals were pooled together to obtain threshold for elevated synchrony detection. We considered *upper* 95% *CI* − *mean* of these pooled data. The threshold for *t*_1−3_ and *h*_2_ used above (upper 95% CI for each individual recording) was replaced with *upper* 95% $CI-mean+\bar{\gamma_{pre(i)}}$, where $\bar{\gamma_{pre(i)}}$ is the average synchrony during the pre-injection epoch for each pair of wires. This newly defined threshold is a fixed constant, it is common for all three different days for all animals. At this new threshold, we observed that all our previous results were statistically significant and were preserved qualitatively. We also observed clear negative correlation of elevated synchrony onset times between the PFC-PFC delta band and PFC-HC theta band (*R* = −0.6276, *p* = 1.028e-3) similar to that at Figure 7. This supports that the dynamics of the synchrony were robustly altered after AMPH injection not only with respect to the changes in variance in the pre-injection epoch but also in absolute terms.

## Discussion

Collectively these data describe the short time scale changes in synchronous network activity that occur immediately following repeated intermittent injections of AMPH. The magnitude and onset of increased synchronous activity changed dynamically as a function of time within a given session, frequency band, and brain region. While a number of changes were observed in the temporal dynamics of synchrony, the most robust and consistent were found in the onset of increased synchrony relative to the pre-injection condition (*t*_1_), where an increase in the onset of delta in the PFC was paralleled by a decrease in the onset of theta between the PFC and HC.

While decreases in synchrony have been observed after psychostimulant self-administration (Reid et al., 2008), in the current study, a brief decrease in synchrony after AMPH was observed in some pairs of LFPs in a minority of animals (see Figure 6E). This effect was infrequent, very shortly-lived, and not statistically significant. The increases in synchrony observed in the current study were larger, were also longer in duration, and thus provide the focus of the current work.

In the current study the positioning of the electrodes in the PFC was, in some cases, ~150 μM. While some of the components of the LFP may have been shared between wires at this distance, the phase-locking levels between the closest sites varied from below 0.2 to above 0.6. This range was consistent with LFPs recorded from much longer distances suggesting that even when recording sites were closest the sources recorded were heterogeneous. Thus, while the interpretation of synchrony levels between closely positioned electrodes is, in general, a complicated matter, the interpretation of the comparison of change in synchrony in the current data is very unlikely to be substantially affected by the inter-electrode distances.

No significant effects of saline injections were observed on the time-course of synchrony in these data, suggesting the injection itself did not alter synchronous dynamics. In contrast to saline injections, AMPH injections significantly altered post-injection synchronous dynamics by affecting both the timing and magnitude of elevated synchrony (relative to the pre-injection epoch) in both brain regions and frequency bands. Alterations in synchrony within the PFC delta band developed, peaked and then dissipated more quickly on Day 1 compared to Days 9 and 23 where the magnitude of these effects were relatively consistent. The delay in the onset, peak, and offset of elevated synchrony in the PFC delta band following repeated AMPH were among the most robust observed in the current study and were consistent with previously observed decreases in average delta power following injection of AMPH (Lapish et al., 2012a). The observed alterations in the time-course of PFC delta likely reflect an impaired ability of disparate neural populations of the PFC to synchronize quickly and possibly contribute some computational function to guide ongoing behavior. Converging evidence establishes that acute high doses of AMPH or repeated injections of low to moderate doses inhibit the firing of PFC neurons (Homayoun and Moghaddam, 2006; Gulley and Stanis, 2010). Progressive decreases in PFC neuronal firing with repeated AMPH is positively correlated with impaired performance of an instrumental responding task and further suggests that repeated AMPH progressively impairs the ability of the PFC to contribute to control of behavior (Homayoun and Moghaddam, 2006). However, the drug-induced changes in PFC are not only limited to neuronal firing. Our results revealed how drug administration affects synchronized neural activity, thus providing another potential mechanism to relate the underlying changes in neurophysiology that facilitate the expression of the altered behavioral phenotype induced by repeated drug use.

The timing of synchronous activity between the PFC and HC, as well as the magnitude of synchrony, were altered over days at both frequency bands. The largest effect observed at the delta band was in the magnitude of peak synchrony (*h*_2_) on Day 1, which was approximately twice that observed on Day 9 and Day 23. It should be noted however that although these delta-band effects were statistically significant, the magnitude of the differences were much smaller than those observed in the theta band between the PFC and HC. This suggests that the AMPH-induced changes in delta may be subordinate to those observed in theta between these structures.

AMPH-evoked changes in the dynamics of theta synchrony between PFC and HC were very prominent. Increases in theta power and synchrony between the PFC and HC following AMPH have been previously observed (Dzirasa et al., 2006; Lapish et al., 2012a). In the current study, however, we observed how the time-course of theta band synchrony between the PFC and HC was substantially modified after each AMPH injection. Along with changes in PFC delta synchrony, these changes were among the most robust observed in the current study. Synchronous activity developed more quickly, was larger in magnitude, and persisted for longer on Days 9 and 23 than on Day 1. Thus, repeated injections of AMPH facilitate synchrony between these two brain regions by both prolonging the interval of elevated synchrony and by boosting synchrony strength. Although repeated AMPH injections significantly altered the dynamics of synchrony when comparing Day 1 with Day 9 and/or Day 23, there was almost no difference between Day 9 and Day 23 except *t*_2_ in PFC-HC theta band, highlighting the persistent nature of these effects over the 2-week cessation period. Increased excitatory outflow from the HC has been shown to powerfully modulate the firing and oscillatory properties of PFC neurons (Hyman et al., 2005, 2011; Jones and Wilson, 2005; Siapas et al., 2005). Moreover, increases in the basal firing rate of HC neurons have been shown to occur following repeated exposure to AMPH, and has been suggested to provide an underlying physiological mechanism for behavioral sensitization (Lodge and Grace, 2008). While a behavioral correlate of sensitization could not be demonstrated in the current study due to the relatively small sample size, our results show that increases in synchrony between the PFC and HC exhibit a pattern consistent with sensitization-like response where it develops faster and persists for longer while the dose of AMPH remains unchanged. This observation could be particularly important to understand how information processing within these circuits is altered with repeated injections of AMPH, and by extension during the development of addiction.

In the current study, progressive changes in PFC-PFC delta synchrony were mirrored by concomitant changes in theta synchrony between the PFC and HC after AMPH injections. Following the first AMPH injection, synchrony within the PFC increased prior to the increases in synchrony observed between the PFC and HC. However, this contingency switched following repeated AMPH exposure such that the increase in synchronous activity between the PFC and HC occurred prior to the onset of synchrony within the PFC. The timing of synchronous network activity may shape the amount and nature of information relayed to different brain areas (Stanley, 2013). Thus, our observation may be evidence that the PFC is more involved in integrating information about AMPH when it is novel following the first exposure, while after many exposures the PFC-HC circuit is recruited and processes the effects of AMPH based on previous histories of exposure. This hypothesis is supported by the finding that HC inactivation blocks the expression of locomotor sensitization following repeated (but not initial) AMPH exposure (Lodge and Grace, 2008).

The observed changes in the time course of synchrony between the PFC and HC could also have important implications for the cognitive functions known to be mediated by these brain regions. Synchronous activity between the PFC and HC can be influenced by changes in dopamine efflux (Benchenane et al., 2010) and is especially important for spatial working memory (Hyman et al., 2005; Jones and Wilson, 2005). The fact that synchrony between the PFC and HC develops more quickly and is more robust after repeated AMPH exposure may reflect a physiological adaptation whereby synchrony can also develop with modest changes in monoamine efflux, such as those observed during spatial working memory (Phillips et al., 2004). Interestingly, accumulating evidence suggests that spatial working memory is *improved* in sensitized rodents (Ito and Canseliet, 2010; Lapish et al., 2012b), while other forms of cognition function, such as flexible decision-making and visual attention, are impaired (Fletcher et al., 2005, 2007). These parallel changes in physiology and behavior may reflect changes in information processing between the PFC and HC after repeated AMPH.

The results of this work illustrate that the time-course of AMPH-evoked phase synchrony within the PFC-HC circuit is dynamic, is influenced by a history of prior AMPH exposures, and involves a persistent (inverse) shift in PFC-HC synchrony onset that might reflect a change in the dominance of information processing between these regions. Given the emerging evidence of the PFC-HC circuit in processing and integrating information about AMPH and other drug experiences (Lodge and Grace, 2008; Wang et al., 2013), our results suggest that the observed rapid transitions in neural synchrony may affect neural communication and influence drug motivated behavior.

### Conflict of interest statement

The authors declare that the research was conducted in the absence of any commercial or financial relationships that could be construed as a potential conflict of interest.
